# What shapes attitudes on gender roles among adolescents in Bangladesh

**DOI:** 10.3389/fpubh.2023.1121858

**Published:** 2023-03-28

**Authors:** Avita J. Streatfield, Md Mahabubur Rahman, Shusmita Khan, M. Moinuddin Haider, Mizanur Rahman, Quamrun Nahar, Kanta Jamil

**Affiliations:** ^1^Independent Consultant, Fitzroy North, VIC, Australia; ^2^International Centre for Diarrhoeal Disease Research, Dhaka, Bangladesh; ^3^Data for Impact, University of North Carolina at Chapel Hill, Chapel Hill, NC, United States

**Keywords:** adolescents, gender role, Bangladesh, attitudes, egalitarian, non-egalitarian

## Abstract

**Background:**

In Bangladesh, large gender differentials exist in outcomes in almost all spheres of life, stemming from conservative norms and attitudes around gender. Adolescence is a crucial period for social-emotional learning that can shape gender norms and attitudes.

**Objective:**

The aim of the paper is to investigate the extent to which adolescents hold egalitarian attitudes toward gender roles, and to examine the factors that influence egalitarian gender attitudes.

**Methods:**

The paper uses data from a nationally representative sample survey of 7,800 unmarried girls and 5,523 unmarried boys ages 15–19 years. Adolescents were considered to have egalitarian attitudes on gender role if they disagreed with all the following four unequal gender role statements with regards to socio-economic participation, while respondents who agreed with any one of the four statements were considered to have non-egalitarian attitudes: (1) It is important that sons have more education than daughters, (2) Outdoor games are only for boys, not girls, (3) Household chores are for women only, not for men, even if the woman works outside the home, and (4) Women should not be allowed to work outside the home. Multivariable linear probability regression analysis was implemented to identify the factors shaping attitudes on gender roles.

**Results:**

Unmarried girls and boys differ hugely in their views on gender roles regarding socio economic participation-girls were much more egalitarian than boys (58 vs. 19%). The multivariate linear probability model results show girls and boys who completed at least grade 10 were 31% points and 15% points more likely to have egalitarian views on gender roles respectively, compared to girls and boys with primary or less education. Having strong connection with parents is associated with having egalitarian views on gender roles among girls but not boys. Adolescents' individual attitude on gender role is highly associated with the views of their community peers for both girls and boys. Girls and boys who had participated in adolescent programs were 6–7% points more likely to have egalitarian attitude than those who were not exposed to these programs. Egalitarian views were also significantly higher, by 5% points among girls and 6% points among boys, who were members of social organizations compared to those who were not. Watching television had positive influence on egalitarian attitudes among girls but not among boys. To create a more egalitarian society, both men and women need to hold progressive attitudes toward gender roles. The interventions must be multilevel, influencing adolescents at the personal, interpersonal, communal, and societal levels.

## 1. Introduction

Throughout history, people have used social learning to acquire adaptive information by copying others' behaviors (1). When the use of social learning results in successful outcomes, individuals continue to undertake these successful behaviors, indicating the development of new social norms (1, 2). Social norms serve as cultural rules that prescribe “acceptable” behaviors, and as such, norms guide individuals' behaviors (2). Social norms may also push an individual to undertake an action that they may not necessarily want to do (3).

Gender presents an additional dimension to the conceptualization of norms. Some scholars believe system of gender includes social norms as one of its several elements, in addition to gender roles, socialization, and gendered power relations (3). Gender can be thought of as a social system in which resources and power are distributed according to whether a person is perceived as masculine or feminine, and as such, gender is also embedded within ideologies and institutions (3–6).

As individuals interact with other members of society or a group, gender norms are enforced and go on to shape attitudes (3, 7). In other words, gender norms are learned and reproduced through social interaction and result in unequal access to resources and freedoms between men and women (3).

Gender attitudes and norms shape the material conditions of people worldwide, from women's participation in the labor force (8), healthcare provision (9), gendered violence (10), and the treatment of the LGBTQIA+ community (11). The literature on gender norms in South Asia specifically also tends to focus on these material impacts. For example, Akter et al. (12) and Psaki et al. (13) describe norms as contributing to a high rate of child marriage in Bangladesh, poverty, lack of agency, climate change insecurity, financial burdens associated with raising girls, and the desire to protect girls from sexual harassment.

Factors shaping attitudes toward gender roles: The literature on gender norms presents several factors that shape the development of gender attitudes among adolescents. A common view is that ideologies are passed down through generations—either through direct interaction or by children observing and modeling behaviors demonstrated by parents (14–19). Cislaghi and Heise (3) maintain that individuals learn gender norms in childhood. Through the process of socialization, individuals internalize norms from their parents and peers, and these norms are then either reinforced or contested in other social institutions like school, the workplace, religion, and media (19–21). Moreover, a systematic review by Kågesten et al. (17) on 82 studies from 29 countries found that gender attitudes among early adolescents (ages 10–14) varied by respondents' sociodemographic characteristics like gender, ethnicity, immigration status, economic class, and age, and these attitudes were further shaped by family and peers.

Girls are more likely to challenge gender inequalities and to hold generally more gender equitable attitudes than boys (17, 19). Literature on the influence of household wealth and social class on gender role attitudes appears to be mixed. In Kågesten et al.'s (17) review, adolescents from higher income backgrounds expressed more equitable gender attitudes, indicating that class may influence opportunities available to young adolescents which in turn may shape gender attitudes. In contrast, a study by Patel et al. (19) did not find any relationship between household wealth and adolescents' gender attitudes. Studies by Bolzendahl and Daniel (22) and Davis and Greenstein (16) hold that education can shape gender role attitudes, and Patel et al. (19) further demonstrated this link with evidence that years of schooling completed had a positive effect on egalitarian gender attitudes of older boys and girls, although this relationship was not found for younger boys and girls (p. 13). Additionally, (17) suggest that schools may “regulate and uphold gender norms” through rules and traditions (like rules around girls' clothing and feminine behavior), as well as disproportionality favoring boys' activities and performance, and teachers reinforcing gender norms.

Kågesten et al.'s (17) study found that no particular form of media appeared to be more influential over shaping gender attitudes (p. 25), although Patel et al. (19) found that all groups of adolescents other than younger boys who used the internet or social media expressed more egalitarian gender role attitudes (p. 13). In line with Patel et al.'s (19) emphasis on media's importance, Webb and Temple (23) and **(author?)** (24) maintain that online media can serve as a platform for adolescents to challenge existing gender norms and explore alternate ones.

In Bangladesh, large gender differentials exist in outcomes in almost all spheres of life—socioeconomic and political participation (25), healthcare (26), property rights (27), and child marriage (28). As Blunch and Das (29) highlight, these forms of gendered inequality stem from Bangladeshi society's conservative gender norms and attitudes. However, there is lack of literature on the prevalence of restrictive gender attitudes in Bangladesh, specifically on the factors that determine gender norms and attitudes in adolescence. Adolescence is a crucial period for social-emotional learning that can shape gender norms and attitudes (30). As such, it is critical to address this gap in the literature because, as scholars like Bhowmik et al. (31) maintain, gendered phenomena like child marriage and adolescent motherhood are prevalent in Bangladesh, limiting women and girls' educational, social, and career opportunities.

This paper's aim is 2-fold: (1) to investigate the extent to which adolescents hold egalitarian attitudes toward gender roles, and (2) to examine the factors—and their degrees of influence— contributing to egalitarian gender attitudes. It explores the association of three sets of factors—(1) individual and contextual factors; (2) peer influence; and (3) social connectivity—with adolescents' attitudes on gender roles. This paper uses data from a nationally representative sample survey of Bangladeshi unmarried boys and girls ages 15–19 and focuses on attitudes toward gender roles on socioeconomic participation that are particularly relevant to adolescents in the Bangladeshi context. Understanding the extent of gender roles' restrictiveness and examining the factors that influence gender attitudes during the teenage years is important for designing appropriate programs and policies that promote a more equitable society for all Bangladeshis.

## 2. Material and methods

This paper uses data from the 2019–20 Bangladesh Adolescent Health and Wellbeing Survey (BAHWS), a nationally representative sample survey of 7,800 unmarried adolescent girls and 5,523 unmarried boys ages 15–19. The BAHWS employed a stratified two-stage sampling to select households. The first stage of sampling involved selecting 728 primary sampling units (PSUs), and the second stage involved selecting households. One hundred households were selected from each PSU using systematic random sampling. In all clusters, all de facto eligible unmarried female adolescents in the 100 selected households were eligible for the questionnaire modules relevant for this paper. Out of these 100 selected households, 70 household were randomly chosen where all de facto unmarried males ages 15–19 were eligible for the interview.

### 2.1. Outcome variable

The dependent variable of the paper is the proportion of adolescents ages 15–19 who have egalitarian attitudes on gender roles. Respondents were considered to have egalitarian attitudes on gender roles if they disagreed with all the following four statements on socio-economic participation. Respondents agreeing with any one of the four statements were considered to have non-egalitarian attitudes toward gender roles.

1. It is important that sons have more education than daughters.
2. Outdoor games are only for boys, not girls.
3. Household chores are for women only, not for men, even if the woman works outside the home.
4. Women should not be allowed to work outside the home.

These four statements were taken from the “Gender-Based Value and Stereotypes Measure” (32) and were specified to Bangladesh's social context, particularly with relation to unmarried adolescents. We excluded 28 out of 7,800 female and 24 out of 5,523 male adolescents from the study who reported “don't know” against any of the four statements.

### 2.2. Explanatory variables

Based on conceptualization, literature review, and information available in the BAHWS, this paper identifies factors that may shape adolescents' egalitarian attitude on gender role. These factors are grouped as: individual characteristics, contextual factors, peer influence, and social connectivity.

1. *Individual characteristics* include the respondents' gender (male or female) and educational attainment. Educational attainment is grouped into three categories: primary school completed or less, secondary schooling incomplete (those who have completed some secondary years of schooling) and completed secondary schooling or higher.
2. *Contextual factors* refer to respondents' immediate surrounding environment, including in which region of Bangladesh they live (western, central, or eastern). Residence captures the urban or rural surroundings in which the respondents live. The measurement of wealth quintile is based on household wealth index and is created using principal component analysis of household assets, categorizing the respondents into in three groups: the two poorest/lowest quintiles, the middle quintile, or the two richest/highest quintiles.
3. *Peer influence* covers the attitudes on gender roles of peers within the household as well as the community that shape adolescents' attitudes. Adolescents' mothers and fathers are considered peers within the household who influence adolescents' attitudes. In this paper, parents are assumed to have egalitarian attitudes when adolescents can connect with them. Adolescents are strongly connected to their mothers/fathers if they stated that they could discuss personal matters with them “always or most of the time.” Adolescents are considered weakly connected to their parents when they expressed that they can only “sometimes or never” discuss personal matters with their parents. It is assumed that adolescents have stronger connection with egalitarian parents. One limitation of this analysis is the absence of survey data to directly measure parents' attitude on gender roles or other attributes that can represent parents' attitudes on gender roles.
4. For a female respondent, her community peers are other unmarried adolescents ages 15–19 in the sampled cluster she resides in; for a male, it is other unmarried boys in the same age group who reside in the same sampled cluster as the respondent. Peers in respondents' clusters of residence were considered “egalitarian” if 60% or more of the unmarried girls/boys ages 15–19 in that cluster disagreed with all four unequal gender role statements, “somewhat egalitarian” if 30–59% disagreed with all four statements, and “non-egalitarian” if <30% disagreed.
5. *Social connectivity* refers to exposures through which adolescents' views can be formed and/or changed. It includes television viewership, internet access, membership in any youth social clubs/organizations, and participation in adolescent-focused programs within the past 3 years.

### 2.3. Statistical analysis

Statistical analysis performed in this paper included univariate analysis of the explanatory variables followed by bivariate analyses between the outcome variables and individual covariates. To examine the association of the covariates with the outcome, we used Rao Scott chi-square test, a design-adjusted version of the Pearson chi-square test used in complex survey data analysis. Finally, the multivariable linear probability model (LPM) was implemented to identify the factors shaping attitudes on gender roles. All the analyses were carried out by incorporating the appropriate survey weight that adjusts for the complex survey design characteristics of the survey.

Logistic regression, a statistical model of the family of Generalized Linear Models, is a common choice to analyze binary outcome in sociological and epidemiological studies. However, researchers often disfavor logistic regression due to the complexity of interpreting odds ratio and its frequent inaccurate interpretations in scientific documents (33, 34). LPM is an alternative that also applies for a binary outcome, particularly when the prevalence is neither low nor high (typically ≥0.2 or ≤ 0.8) (35–38). A primary advantage of LPM is that its coefficients are interpretable as probabilities. Although applying LPM while dealing with binary outcome is widely recommended for impact evaluation analysis, it also applies in our case. We used LPM for the simplicity of its coefficients to interpret. We also provide logistic regression findings to cross-check the results in different models.

## 3. Results

As shown in Table 1, 35.9% of all unmarried adolescents ages 15–19 expressed egalitarian views, where they disagreed with all the four statements on unequal gender roles. However, unmarried girls and boys differ hugely in their views on gender roles. Girls tend to be more egalitarian than boys, with 58.3% of all females compared to 18.6% of the males disagreeing with the four unequal gender statements regarding socioeconomic participation. Non-egalitarian gender attitudes were more extreme among boys than girls, with 37.7% of boys disagreeing with only one or none of the unequal gender role statements, compared to only 12.2% of the girls.

**Table 1:** Percentage of respondents disagreeing with number of unequal gender role statements.

	**Egalitarian**	**Non-egalitarian**					
	**All four statements**	**Three statements**	**Two statements**	**One statement**	**None**	**Total (%)**	*N* ^*^
All unmarried adolescents	35.9	20.2	17.3	14.7	11.9	100.0	13,268
Unmarried female adolescents	58.3	18.7	10.7	7.0	5.2	100.0	7,770
Unmarried male adolescents	18.6	21.3	22.4	20.7	17.0	100.0	5,499

* Respondents who answered “do not know” to any of the four statements are excluded.

**Figure 1:**
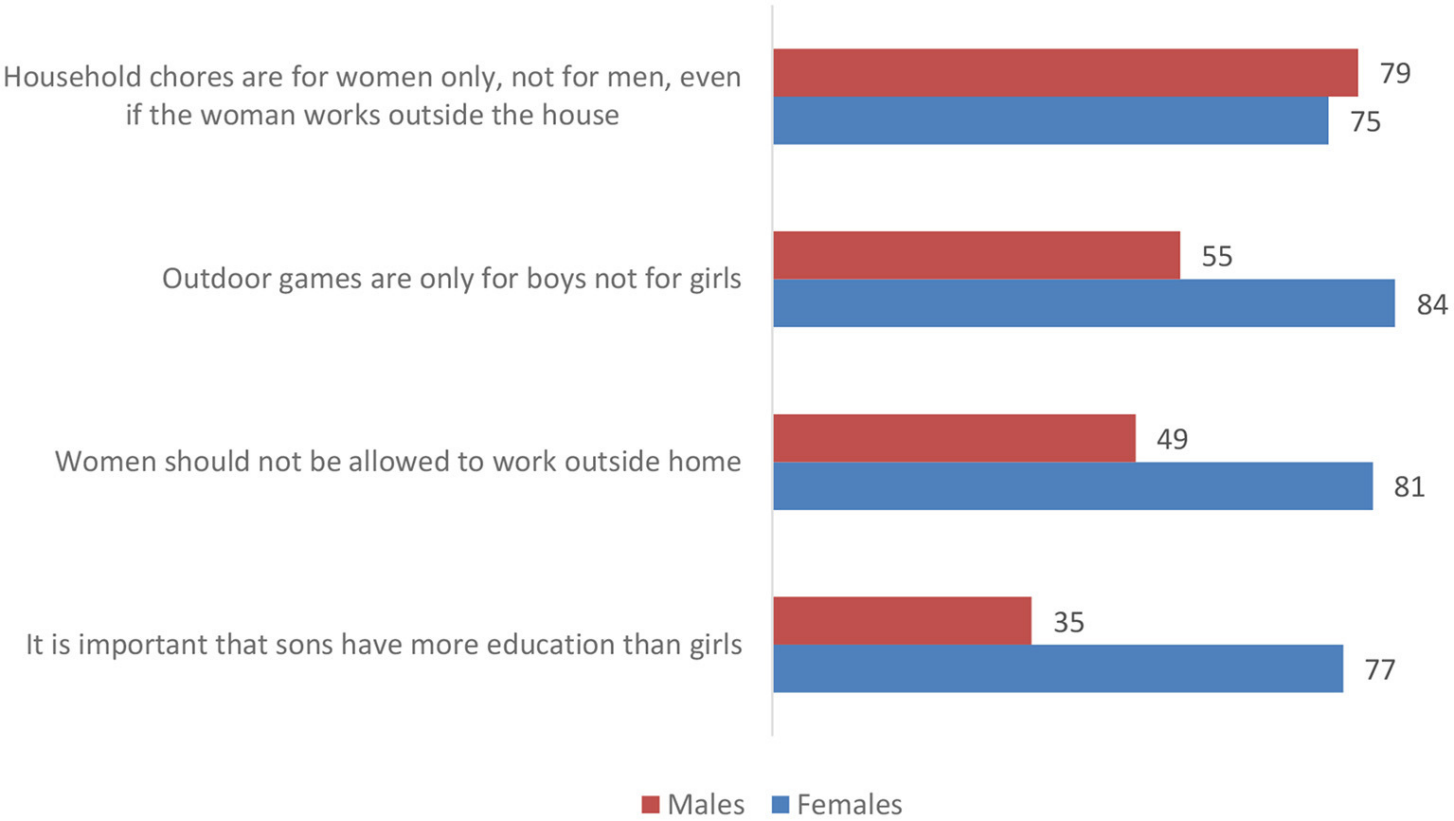
Percentage (%) of unmarried female and male adolescents (ages 15–19) disagreeing with statements on unequal gender roles.

Figure 1 illustrates the percentage of girls and boys who disagreed with each of the four statements on unequal gender roles. Only one third of the boys disagreed with the statement “it is important that sons have more education than girls,” and around half disagreed with “women should not be allowed to work outside the home” and “outdoor games are only for boys.” In contrast, around four out of five (77–84%) adolescent girls disagreed with each of these three statements. Interestingly, both boys and girls tended to align on their disagreement with “household chores are for women only, not for men, even if the woman works outside the home” with 75% of girls and 79% of boys disagreeing with the statement.

Given the large difference in attitudes on gender roles, this paper examines background characteristics and factors influencing gender role attitudes separately for girls and boys.

Table 2 shows that girls tended to be more well-educated than boys. Nearly one-fifth of males only had a primary school level education or less, compared to just <1 tenth of girls. A higher proportion of girls also had completed secondary schooling or higher (35.5% of females vs. 30.3% of males). Over two thirds of girls and boys lived in rural areas.

**Table 2:** Background characteristics of unmarried female and male adolescents ages 15–19.

**Characteristics of adolescents**	**Unmarried females**		**Unmarried males**	
	**Distribution (%)**	***n***	**Distribution (%)**	***n***
**Individual and contextual factors**				
**Educational attainment**				
Primary completed or less	8.3	628	18.7	1,014
Secondary incomplete	56.3	4,277	51.0	2,761
Secondary completed or higher	35.5	2,695	30.3	1,640
**Household wealth quintile**				
Lowest two quintiles	32.9	2,501	38.4	2,079
Middle quintile	22.0	1,673	20.7	1,120
Highest two quintiles	45.1	3,427	40.9	2,215
**Residence**				
Urban	32.1	2,441	28.7	1,554
Rural	67.9	5,160	71.3	3,861
**Region**				
Western	29.9	2,275	37.2	2,014
Central	38.7	2,942	36.3	1,963
Eastern	31.3	2,383	26.5	1,437
**Peer influence**				
**Connectedness with mother**				
Can always/most times discuss personal matters with mother	54.8	4,166	20.5	1,111
Can never/sometimes discuss personal matters with mother	45.2	3,435	79.5	4,303
**Connectedness with father**				
Can always/most times discuss personal matters with father	11.8	900	13.2	714
Can never/sometimes discuss personal matters with father	88.2	6,700	86.8	4,700
**Community peers' attitudes on gender roles**				
Non-egalitarian	9.6	727	77.7	4,196
Somewhat egalitarian	40.7	3,090	17.1	922
Egalitarian	49.8	3,779	5.2	281
**Social connectivity**				
**Watches TV at least once a week**				
Yes	74.4	5,653	78.5	4,251
No	25.6	1,947	21.5	1,163
**Accesses internet at least once a week**				
Yes	22.2	1,685	47.4	2,569
No	77.8	5,915	52.6	2,846
**Member of any youth club/social organization**				
Yes	14.0	1,065	22.2	1,201
No	86.0	6,536	77.8	4,213
**Participation in any program focused on adolescents (anytime in the last 3 years)**				
Yes	11.1	847	5.6	410
No	88.9	6,753	94.4	5,004
**Total**	**100**	**7,600**	**100**	**5,414**

Boys tended to experience low levels of connectedness with their parents, with only 20 and 13% of the boys stating they could discuss personal matters with their mother and father. Similarly, very few girls stated that they could discuss personal matters with their fathers (12%), but over half could do so with their mothers, indicating more closeness. Girls' and boys' surrounding peers exhibited clear gendered differences in gender attitudes. Half of the girls lived in communities where her peers (other girls of same age group) held egalitarian attitudes on gender roles. Boys may be living in the same community as girls, but only 5.2% of the boys had community peers who had egalitarian views on gender roles.

The majority of both girls and boys watched television at least once a week (about 75% of both groups). These proportions, however, changed for internet access. Nearly half of all boys used the internet at least once a week, compared to less than one fourth of the girls. Boys were slightly more active than girls in joining youth clubs and social organizations, with 22.2% of boys being active members compared to 14% of girls. Participation in adolescent programs were low for both genders, albeit slightly higher for girls-−11.1% of girls had participated in any adolescent-focused program in the last 3 years, and only 5.6% of boys did the same.

### 3.1. Bivariate analysis

As seen in Table 3, bivariate analysis indicates that all variables under individual characteristics and contextual factors, peer influence, and social connectivity were statistically significant for unmarried girls. For boys, all were significant other than region and connectedness with their mother/father. The proportion of girls and boys with egalitarian views increased with the level of educational attainment. Household wealth also had a statistically significant impact on egalitarian views, where the highest proportion of egalitarian girls and boys were in the highest 40% wealth quintile. Girls and boys living in urban areas also were more egalitarian than those in rural areas, and girls living in the western region were more egalitarian than girls in the central and eastern regions. For boys, the differences between regions were not statistically significant.

**Table 3:** Percentage of adolescents (ages 15–19) who have egalitarian attitudes on gender role by background characteristics.

**Characteristics of adolescents**	**Unmarried females**		**Unmarried males**	
	**Egalitarian (%)**	***X***^2^ **test (*****P*****-value)**	**Egalitarian (%)**	***X***^2^ **test (*****P*****-value)**
**Individual and contextual factors**				
**Educational attainment**				
Primary completed or less	28.6	<0.001	7.9	<0.001
Secondary incomplete	54.3		17.2	
Secondary completed or higher	72.4		27.9	
**Household wealth quintile**				
Lowest two quintiles	47.5	<0.001	13.1	<0.001
Middle quintile	55.7		14.9	
Highest two quintiles	68.1		25.8	
**Residence**				
Urban	66.2	<0.001	24.2	<0.001
Rural	55		16.5	
**Region**				
Western	63	<0.001	18.5	0.966
Central	58.8		18.9	
Eastern	54.2		18.6	
**Peer influence**				
**Connectedness with mother**				
Can always/most times discuss personal matters with mother	63.2	<0.001	19.7	0.410
Can never/sometimes discuss personal matters with mother	53.1		18.4	
**Connected with father**				
Can always/most times discuss personal matters with father	67.2	<0.001	19.7	0.508
Can never/sometimes discuss personal matters with father	57.5		18.5	
**Community peers' attitude on gender roles**				
Non-egalitarian	40.3	<0.001	14.0	<0.001
Somewhat egalitarian	52.5		29.9	
Egalitarian	67.1		50.3	
**Social connectivity**				
**Watches TV at least once a week**				
Yes	62.6	<0.001	19.6	<0.003
No	47		15.4	
**Accesses internet at least once a week**				
Yes	70.9	<0.001	21.9	<0.001
No	55.1		15.7	
**Member of any youth club/social organization**				
Yes	69.2	<0.001	27.7	<0.001
No	56.9		16.1	
**Participation in any program focused on adolescents (anytime in the last 3 years)**				
Yes	69.2	<0.001	28.3	<0.001
No	57.3		18.1	
**Total**	**58.6**		**18.7**	

Girls who felt more connected with their mothers and fathers were more egalitarian, whereas there was no statistically significant difference for boys' connectedness with their mothers nor fathers. Girls and boys who had egalitarian peers also tended to be more egalitarian.

Exposure to TV and the internet had a significant effect—both girls and boys who watched TV and accessed the internet expressed more egalitarian attitudes. Membership in youth clubs and organizations also had an effect, wherein girls and boys who were active in these groups were also more egalitarian. The same effect was found for participation in adolescent programs for both girls and boys.

### 3.2. Multivariate analysis

Table 4 shows estimates of the change in the probability of having egalitarian attitude on gender roles among girls according to individual, contextual, peer influence, and social connectivity factors.

**Table 4:** Linear probability regression based adjusted coefficient of egalitarian attitudes on gender roles among unmarried females ages 15–19.

**Characteristics of adolescents**	**Coefficient**	**95% CI**
**Individual and contextual factors**		
**Educational attainment (ref: Primary completed)**		
Secondary incomplete	0.21^**^	[0.16, 0.25]
Secondary completed or higher	0.34^**^	[0.30, 0.39]
**Household wealth quintile (ref: Lowest two quintiles)**		
Middle quintile	0.04^*^	[0.01, 0.07]
Highest two quintiles	0.10^**^	[0.07, 0.13]
**Residence (ref: Rural)**		
Urban	0.05^**^	[0.02, 0.07]
**Region (ref: Western)**		
Central	−0.03^*^	[−0.06, −0.01]
Eastern	−0.06^**^	[−0.09, −0.03]
**Peer influence**		
**Connectedness with mother (ref: Can never/sometimes discuss**		
**personal matters with mother)**		
Can always/most times discuss personal matters with mother	0.05^**^	[0.03, 0.08]
**Connected with father (ref: Can never/sometimes discuss**		
**personal matters with father)**		
Can always/most times discuss personal matters with father	0.06^**^	[0.02, 0.09]
**Community peers' attitude on gender roles**		
**(ref: Non-egalitarian)**		
Somewhat egalitarian	0.09^*^	[0.00, 0.17]
Egalitarian	0.17^**^	[0.09, 0.24]
**Social connectivity**		
**Watches TV at least once a week (ref: No)**		
Yes	0.08^**^	[0.05, 0.11]
**Accesses internet at least once a week (ref: No)**		
Yes	0.03	[−0.00, 0.06]
**Member of any youth club/social organization (ref: No)**		
Yes	0.05^**^	[0.02, 0.08]
**Participation in any program focused on adolescents**		
**(anytime in the last 3 years) (ref: No)**		
Yes	0.07^**^	[0.03, 0.11]
Constant	0.08^*^	[0.00, 0.15]
Observations	7,596	

* p <0.05.

** p <0.01.

Estimates from the fully adjusted regression model show that the probability of having egalitarian attitudes on gender role increased with educational attainment and household wealth quintile. Girls with some secondary schooling and those who completed secondary schooling or more were 21% points (ppt) at *p* < 0.01 and 34 ppt at *p* < 0.01, respectively, more likely to have egalitarian attitudes on gender role compared to girls with primary complete or lesser educational attainment. The probability of having egalitarian views was 4 ppt (at *p* < 0.05) higher among girls from households in the middle wealth quintile and 10 ppt (at *p* < 0.01) greater among those from households in the top two wealth quintiles, compared to girls from the bottom two wealth quintile households. Urban girls were 5 ppt (at *p* < 0.01) more likely to have an egalitarian attitude on gender norms than those in rural areas. Unmarried adolescent girls who lived in the central and eastern regions of Bangladesh were less likely to have egalitarian views (−3 ppt at *p* < 0.05 and −6 ppt at *p* < 0.01, respectively) compared to those from the western region.

Peer influence factors were significantly associated with attitudes on gender roles after controlling for other influencing factors. Girls who were more connected with their mothers and fathers had a higher probability of having egalitarian views (5 ppt at *p* < 0.01 and 6 ppt at *p* < 0.01, respectively) than those who were not connected with their mothers/fathers. Community peers' attitudes on gender roles also had a high influence on individual attitude on gender roles. Girls who had egalitarian community peers were 17 ppt (at *p* < 0.01) more likely and those with somewhat egalitarian community peers were 9 ppt (at *p* < 0.05) more likely to have egalitarian attitude compared to girls who had non-egalitarian community peers.

Among the social connectivity factors, access to television viewing, membership in social organizations/clubs, and participation in programs for adolescents were significantly associated with having egalitarian attitudes on gender roles among unmarried adolescent girls. Those who watched TV at least once a week were 8 ppt (at *p* < 0.01) higher probability of being egalitarian compared to those having no or less exposure to TV viewing. Internet access did not have a significant influence on egalitarian attitudes. The probability of having egalitarian views on gender role was 5 ppt (at *p* < 0.01) higher among those who were members of social organizations/clubs for youths than non-members. Participation in adolescent-focused programs increased the likelihood of being egalitarian by 7 ppt (at *p* < 0.01) compared to those who did not.

Table 5 displays the results of the same multivariate analysis for unmarried boys ages 15–19. Higher educational attainment increased the likelihood of being egalitarian but to a lesser degree compared to the influence it has among adolescent girls. Boys who completed at least secondary schooling were 15 ppt (at *p* < 0.01) more likely to have egalitarian attitude on gender roles compared to those with primary complete or less educational attainment. The probability of being egalitarian was 6 ppt (at *p* < 0.01) higher among boys who had some secondary schooling compared to those who did not attend secondary school. Only those who came from the highest two wealth quintile households had a higher likelihood of being egalitarian (7 ppt at *p* < 0.01) compared to those from the two lowest wealth quintile households. Urban and rural boys had similar attitudes on gender roles. Also, there was no variation on egalitarian views among boys by region.

**Table 5:** Linear probability regression based adjusted coefficient of egalitarian attitudes on gender roles among unmarried males ages 15–19.

**Characteristics of adolescents**	**Coefficient**	**95% CI**
**Individual and contextual factors**		
**Educational attainment (ref: Primary completed)**		
Secondary incomplete	0.06^**^	[0.04, 0.08]
Secondary completed or higher	0.15^**^	[0.12, 0.18]
**Household wealth quintile (ref: Lowest two quintiles)**		
Middle quintile	0.00	[−0.02, 0.03]
Highest two quintiles	0.07^**^	[0.05, 0.10]
**Residence (ref: Rural)**		
Urban	0.02	[−0.01, 0.04]
**Region (ref: Western)**		
Central	0.00	[−0.03, 0.02]
Eastern	0.00	[−0.03, 0.02]
**Peer influence**		
**Connectedness with mother (ref: Can never/sometimes discuss**		
**personal matters with mother)**		
Can always/most times discuss personal matters with mother	−0.01	[−0.04, 0.02]
**Connected with father (ref: Can never/sometimes discuss**		
**personal matters with father)**		
Can always/most times discuss personal matters with father	−0.01	[−0.04, 0.02]
**Community peers' attitude on gender roles**		
**(ref: Non-egalitarian)**		
Somewhat egalitarian	0.14^**^	[0.08, 0.20]
Egalitarian	0.32^**^	[0.22, 0.42]
**Social connectivity**		
**Watches TV at least once a week (ref: No)**		
Yes	0.02	[−0.01, 0.05]
**Accesses internet at least once a week (ref: No)**		
Yes	−0.01	[−0.03, 0.01]
**Member of any youth club/social organization (ref: No)**		
Yes	0.07^**^	[0.04, 0.10]
**Participation in any program focused on adolescents**		
**(anytime in the last 3 years; ref: No)**		
Yes	0.06^*^	[0.01, 0.11]
Constant	0.01	[−0.03, 0.04]
Observations	5,399	

* p <0.05.

** p <0.01.

Regarding peer influences, connectedness with mother or father had no association with egalitarian views on gender role among boys. However, community peer attitudes had a huge influence on boys' individual attitude on gender roles. Boys whose community peers had egalitarian attitudes were 32 ppt (at *p* < 0.01) more likely to have egalitarian attitudes.

Among the social connectivity factors, being members of social organizations/youth clubs and exposure to programs for adolescents significantly increased the probability of having egalitarian attitudes on gender roles among adolescent boys (7 ppt at *p* < 0.01 and 6 ppt at *p* < 0.05, respectively).

The analysis performed in Tables 4, 5 was repeated using multivariable logistic regressions and the results are presented in Appendix. The results remained similar and in the same direction. The only difference that occurred was logistic regression found access to internet a statistically significant (*p* < 0.049) correlate of egalitarian gender attitudes which was not statistically significant in the LPM analysis (*p* < 0.066). Although that is different based on statistical significance, the difference in predicted probabilities of being egalitarian between those who were exposed to internet and who were not is only 3% points.

## 4. Discussion

Inequity in health status and access to health services by gender are influenced by individual and community attitudes on gender roles. Health practices are also negatively affected by traditional gender norms. For example, in a society with restrictive attitudes toward masculinity and femininity, rigid gender norms will continue to uphold public health concerns like child marriage and adolescent motherhood among girls, and relatively high health risk behavior among boys. In order to achieve equitable health improvements, it is crucial that gender norms are examined and reconstructed.

The study examined the influence of selected sets of factors namely individual (gender and years of schooling), contextual (wealth quintile, regional variation, and urban-rural residence), peer-influence (connectedness with parents, community peers' attitude on gender role), and social-connectivity (TV watching, access to internet, membership in social organization, and participation in adolescents' program) on adolescents' attitudes on gender roles in Bangladesh. The study used data from a nationally representative sample survey of over 13,000 unmarried adolescents ages 15–19. An adolescent being female, having 10 or higher years of schooling, belonging to households in the upper two wealth quintiles, are likely to have egalitarian attitude compared to his/her counterpart. Having strong connections with parents is associated with having egalitarian views on gender role among girls but not boys. Adolescents' attitudes on gender role are highly associated with the views of their community peers. Regarding the influence of social-connectivity, girls and boys who have participated in adolescent programs and/or are members of social organizations are more likely to have egalitarian attitudes. Watching television has positive influence on egalitarian attitudes among girls but not among boys.

This study found that girls are three times more likely to have egalitarian views on gender role than boys. This finding is in line with other studies where girls held more egalitarian attitudes (39–41). Girls may tend to be more egalitarian because they are the primary victims of gender discrimination upheld by the patriarchy, and thus have more to gain from favoring an egalitarian society (39, 42). Boys hold more social power as members of a higher status group and may want to maintain their position in the social hierarchy (39, 43).

Another possibility for the boys' conservative attitudes is that gender stereotypes tend to be more rigid for men and boys. During early adolescence, gender attitudes become more ingrained, but they can become more flexible over time if a child's environment is sufficiently egalitarian (39, 44–46). However, boys tend to be limited by more rigid masculine norms, as attributes associated with masculinity tend to be less flexible than those associated with femininity (46–48). Males are more harshly “punished” by way of social critique or ostracization for deviating from traditional masculine stereotypes. Such traditional norms include concepts such as the “boys don't cry” mentality which discourages men for expressing emotional distress and seeking help for emotional problems (46, 49).

As such, when designing interventions to foster egalitarian attitudes, one needs to keep in mind that girls are generally more egalitarian than boys and the concept of gender formation is carried out from an early age (50). A study conducted in the 1980's (51) demonstrated that boys and girls within the same 3-year age range showed different communication styles, participated in different activities, played more often with same-gender friends, and tended to avoid making friends with the opposite gender. A 2020 study discovered that gender stereotypes become particularly rigid for boys at an early age, with boys ages 4–9 already internalizing gender roles (46). Interventions must therefore aim to expand boys' attitudes beyond traditional masculine norms of reputation, strength, or sexual prowess. They must instead relate masculinity with more egalitarian attributes like open-mindedness and compassion for others (52, 53). These approaches might follow the example of Park et al.'s (52) program, which encouraged boys toward gender equitable views like open communication, non-violent conflict resolution, and respect for others.

Boys can learn how they will also benefit from more progressive attitudes and free themselves from restrictive traditional norms of masculinity. For example, traditional notions of masculinity pressure men to be the “breadwinners,” or the principal providers of money for a household (54). However, if boys are socialized to understand that all genders should have equal opportunities for education and work outside of the home, they may face reduced pressure.

Keeping both girls and boys in school is critical for development of egalitarian attitudes, as adolescents are socialized by classroom peers (39, 53). Through the “hidden curriculum,” adolescents learn gender stereotypes by internalizing subtle messages about what is expected of each gender (46, 55). The hidden curriculum plays a role in socializing adolescents to be more egalitarian, as our findings show that both girls and boys who complete secondary schooling are more egalitarian than their less-educated counterparts. Ensuring that adolescents, irrespective of gender, pass secondary schooling should be a key priority.

In Bangladesh, school dropouts among both adolescent boys and girls are substantial. According to the 2017–18 Bangladesh Demographic and Health Survey, 45% of adolescents ages 15–19 are married. School discontinuation is as high as 83% among married adolescent girls (56). Thus, married adolescents have less educational attainment than unmarried girls of same age (56). The Female Secondary School Stipend Project provided cash stipends to retain girls in secondary school (57) and it had a positive impact on secondary school retention among girls and increased age of marriage (58). However, one third of girls are still marrying before turning 16 (59) and dropping from school before completing secondary education. Additional incentives like employment opportunities and free vocational training for those completing secondary education may be considered to encourage girls to complete secondary schooling.

In terms of educational attainment, adolescent boys are lagging behinds girls of same age group. For example, according to the 2017–18 Bangladesh Demographic and Health Survey, 83% of the girls ages 15–19 have at least some secondary schooling compared to 74% of the boys (60). School dropout rates among unmarried boys is 28%. This high dropout rate among boys is primarily due to issues of poverty and lack of interest—over 90% of boys dropped out due to financial constraints or high cost of schooling (51%) or lack of interest in the curriculum (57%) (56). As a means of reducing dropout rates amongst boys who may be dropping out to begin working to support their families, financial incentives may be considered. Approaches like the Female Secondary School Stipend Project may be introduced to keep poorer boys in school. Further research is needed to investigate boys' lack of interest in school curricula. Once the reasons for disinterest are uncovered, steps can be taken to modify curricula and adapt modes of learning to suit students' learning needs.

Schools must be used as a platform for development of egalitarian attitudes. School-based education programs have been found to create more progressive attitudes toward girls and women, as well as less agreement with traditional masculine norms (61). These programs would benefit from taking a gender-transformative approach. Gender-transformative programs address harmful aspects of masculinity, allowing males to challenge and critique discriminatory norms and roles and resist masculine stereotypes that have a negative impact on health and wellbeing (49, 52, 62). International examples for school-based education can be seen in Australia's Breaking the Man Code workshops, which aimed to challenge masculinities with young men to encourage them to seek help for emotional problems and reduce suicide risk (49). Similarly, the WiseGuyz Program in Canada engaged boys to reflect on, and challenge, gender-inequitable attitudes and behaviors (62).

A notable Bangladeshi project is the Generation Breakthrough project by UNFPA, initiated in 2012 with support from Embassy of the Kingdom of the Netherlands. This project covered around 140,000 adolescents ages 10–19 from 300 secondary schools, 50 madrasas (Islamic schools), and 150 adolescent clubs across Bangladesh (63). The gender-related key achievement of this project includes: (1) production and approval of Gender Equity Movement in School (GEMS) curricula by the NCTB (National Curriculum and Text Book Board) authority; (2) production and approval of SRHR materials by the Directorate of Secondary and Higher Education (DSHE) under MOE (ministry of education); (3) production and distribution of around 100,000 GEMS dairies in district level education offices; and (4) ground work created for exercising broader influence around the use of GEMS in national school curricula (64). The project's midterm evaluation shows that though there has been positive change against gender stereotypes by the students, gender differential on perception still exists significantly. For instance, boys were 1.7 times (OR = 1.7) more likely to believe that a “husband can beat his wife sometimes,” and four times (OR = 4.0) more likely to believe that “it is girl's fault if a male student or teacher sexually harasses her” (65). Furthermore, the qualitative evaluation identified notable challenges like a lack of clarity in conceptualization of gender roles by the Gender Promoters while facilitating sessions and lack of engagement among school management committees, parents, and students in the Madrasas (64).

While challenging gender inequities amongst boys is crucial, it is also important for girls' inclusion in various activities to be prioritized. Schools and other social platforms can be used to showcase girls' participation and successes in sports, debates, and cultural activities like dancing and literary competitions. Mass media also plays a role in highlighting girls' accomplishments in these areas, as doing so will promote an enabling environment to support girls in participating in activities alongside boys.

In terms of peer influence on gender attitudes, this study's findings show that boys were less connected with both their mothers and fathers compared to girls. Our study did not evaluate the gender attitudes held by adolescents' parents, which may be a useful direction in future research. Parental attitudes are a significant predictor of children's attitudes toward gender, as parents perform practices in the household that reflect their gender attitudes (39, 53). In turn, children construct similar attitudes to their parents' (66).

During adolescence, peers may exert an even greater influence on behavior than parents (53). In this study, boys and girls ages 15–19 who lived in the same cluster as the respondent were considered to be the community peer group of the respondent. Whilst girls with egalitarian peers are 17% points more likely to be egalitarian than those with conservative peers, boys were even more influenced by their peers—boys with egalitarian peers were about 32% points more likely to have egalitarian attitudes than those with conservative peers. This may be because egalitarian boys tend to behave in ways that go beyond traditional masculine norms, and boys' peers exert a strong influence on individual attitudes (53). Jewell et al.'s (67) study, for example, found that adolescents tend to value peer approval, so an individual's gender attitudes and behavior tend to match those of their peers.

The study findings show that attendance in adolescent-focused youth programs had a significant impact on both boys' and girls' egalitarian attitudes toward gender norms. Youth programs had a particularly strong effect on boys: only 5.6% of boys attended adolescent programs, but those who did were 7% points more likely to have egalitarian views on gender roles than those who did not. It is possible, however, that the boys attending these programs were already egalitarian-leaning, implying a selectivity bias. Because fewer boys attended these programs than girls, more research is needed to explore how boys' participation can be increased.

The strong influence of these programs on boys implies the need for both male- and female-focused interventions. Adolescent health programs in Bangladesh tend to focus predominantly on girls, paying little specific attention to male-specific issues (68). It is important that these programs aim to reach boys, particularly because early adolescence marks a time when boys' interpersonal interactions are centered around their friendships with other boys (69). During this time, male adolescents also tend to “police” others' adherence to gender norms as a way of socializing other boys to act as traditionally masculine as possible (69).

To shift gender attitudes, all three factors—individual and contextual factors, peer influence, and social connectivity—must be considered. Interventions must be multilevel to target the personal, interpersonal, communal, and societal levels (52). It is particularly important that interventions take advantage of existing forums and institutions by, for example, taking place at schools, social clubs, or organizations (39, 68). Interventions should aim to not only influence individual adolescents' attitudes but the attitudes of adolescents' parents, peers, and broader community members (39, 52). As a starting point, interventions can include male peer support groups as “safe spaces” for boys to discuss the challenges of traditional masculinity (52, 70). The overarching goal should be to shift boys' notions of masculinity by, for example, guiding boys on strategies for non-violent conflict resolution (52).

Media channels can also be leveraged to bring about egalitarian attitudes. The popular MTV reality show *16 and Pregnant* followed the lives of teenage girls who were pregnant. An analysis suggested that the show was associated with a reduction of teenage pregnancy in the US by up to one-third, illustrating the potential for media to facilitate behavior change (71). Targeted messaging by TV shows aimed at adolescents may further cultivate egalitarian attitudes. Similarly, egalitarian attitudes can be further reinforced at casual social gatherings like concerts and religious sermons. Discussions of gender issues could be integrated into the agendas of such events, further reinforcing egalitarian attitudes.

Changes in attitudes toward gender norms will not occur overnight. Multilevel approaches must be taken over time, and their impacts on adolescents' attitudes closely monitored. By considering which attitudes are changing and which are not, we will be able to track progress toward a more egalitarian society. Norms that remain rigid may need special attention and further research. There is dearth of evaluations of programs targeting adolescents in general. Rigorous evaluation and well-designed learning agenda should be an integral part of program designs aiming to shift attitudes and practices related to gender roles among adolescents so successful interventions can be taken to scale.

## 5. Limitations

The study is based on secondary analysis of a national survey and thus is limited by data available from the survey. The paper defined having egalitarian attitude on gender role based on responses to four questions that have relevance to Bangladeshi culture and its gender attitude norms. This definition of egalitarian attitude may be limited and may not hold across other cultures and societies. In addition, attitude on gender roles can change and shape over time and is influenced by changes in environment and social connectivity, which this study is unable to capture. While the survey provided rich data on some factors, it lacked in-depth information on certain variables. It is well-established that peer influence from within the household is an important factor impacting adolescents' egalitarian attitudes on gender roles. There was no direct measure of adolescents' parents' (who are considered in this study as influencing peers) attitudes on gender roles. Among the social connectivity factors, more information on types of programs watched on TV and information viewed on internet would have been useful to explain the results. Also, information on the intensity of participation in program for adolescents and whether these programs addressed gender issues were not available to enrich the analysis. Despite these limitations, the study provides useful and significant guidance on programmatic steps to be considered to increase egalitarian attitudes on gender roles among both boys and girls.

## Data availability statement

The datasets presented in this study can be found in online repositories. The names of the repository/repositories and accession number(s) can be found at: https://dataverse.unc.edu/dataset.xhtml;jsessionid=77a20841a735135e04ab7cadf42e?persistentId=doi%3A10.15139%2FS3%2FDVEI9A&version=&q=&~fileTypeGroupFacet=%22Document%22&fileTag=&fileSortField=&fileSortOrder.

## Ethics statement

The studies involving human participants were reviewed and approved by the Ethical Review Committee of the ICDDR,B. Written informed consent to participate in this study was provided by the participants' legal guardian/next of kin.

## Author contributions

KJ conceptualized and directed the design of the analysis and drafted parts of the paper. AS conducted the literature review and was the primary drafter of the manuscript. MMR and MH performed the statistical analysis and contributed to writing the methods. SK provided support in literature review and writing a part of the discussion as well as reviewing the paper. MR contributed to refining the statistical analysis. MR and QN reviewed iterations of the draft manuscript and contributed to refining the discussion of the study findings. All authors reviewed and contributed to finalization of the manuscript and approved the final version of the article.
