# Supplementary material for: What shapes attitudes on gender roles among adolescents in Bangladesh
Source: Front Public Health. 2023 Mar 28;11:1121858. doi: 10.3389/fpubh.2023.1121858 (PMC10088519; doi:10.3389/fpubh.2023.1121858)
Supplement: Supplementary file 1 [file Table_1.docx]

**Appendix**

**Table 4A. Logistic regression based adjusted odds ratio of egalitarian attitudes on gender roles among unmarried females ages 15–19**

| **Characteristics of adolescents** |  | |
| --- | --- | --- |
|  | **AOR** | **95% CI** |
| ***Individual and contextual factors*** |  |  |
| **Educational attainment (ref: Primary completed)** | Reference |  |
| Secondary incomplete | 2.52^**^ | [2.04,3.12] |
| Secondary completed or higher | 4.75^**^ | [3.77,6.00] |
| **Household wealth quintiles (ref: Lowest two quintiles)** | Reference |  |
| Middle quintile | 1.18^*^ | [1.02,1.37] |
| Highest two quintiles | 1.56^**^ | [1.35,1.79] |
| **Residence (ref: Rural)** | Reference |  |
| Urban | 1.26^**^ | [1.11,1.44] |
| **Region (ref: Western)** | Reference |  |
| Central | 0.86^*^ | [0.76,0.97] |
| Eastern | 0.76^**^ | [0.66,0.87] |
| *Peer influence* |  |  |
| **Connectedness with mother (ref: Can never/sometimes discuss personal matters with mother)** | Reference |  |
| Can always/most times discuss personal matters with mother | 1.27^**^ | [1.13,1.43] |
| **Connected with father (ref: Can never/sometimes discuss personal matters with father)** | Reference |  |
| Can always/most times discuss personal matters with father | 1.33^**^ | [1.11,1.59] |
| **Community peers’ attitude on gender roles (ref: Non-egalitarian)** | Reference |  |
| Somewhat egalitarian | 1.47^*^ | [1.01,2.12] |
| Egalitarian | 2.13^**^ | [1.51,2.99] |
| ***Social connectivity*** |  |  |
| **Watches TV at least once a week (ref: No)** | Reference |  |
| Yes | 1.43^**^ | [1.26,1.63] |
| **Accesses internet at least once a week (ref: No)** | Reference |  |
| Yes | 1.17^*^ | [1.00,1.36] |
| **Member of any youth club/social organization (ref: No)** | Reference |  |
| Yes | 1.28^**^ | [1.09,1.51] |
| **Participation in any program focused on adolescents (anytime in the last 3 years) (ref: No)** | Reference |  |
| Yes | 1.39^**^ | [1.16,1.67] |
| Observations | 7596 |  |
| **Had accesses to internet at least once a week** | Predicted  probabilities | p-value |
| Yes | 0.61 | - |
| No | 0.58 | - |
| P(Yes) – P(No) | 0.03 | 0.049 |

* *p* < 0.05, ** *p* < 0.01

**Table 5A. Logistic regression based adjusted odds ratio of egalitarian attitudes on gender roles among unmarried males ages 15–19**

| **Characteristics of adolescents** | **Model 3** | |
| --- | --- | --- |
|  | **AOR** | **95% CI** |
| ***Individual and contextual factors*** |  |  |
| **Educational attainment (ref: Primary completed)** | Reference |  |
| Secondary incomplete | 2.00^**^ | [1.51,2.66] |
| Secondary completed or higher | 3.54^**^ | [2.66,4.70] |
| **Household wealth quintiles (ref: Lowest two quintiles)** | Reference |  |
| Middle quintile | 1.06 | [0.83,1.37] |
| Highest two quintiles | 1.70^**^ | [1.38,2.09] |
| **Residence (ref: Rural)** | Reference |  |
| Urban | 1.09 | [0.92,1.30] |
| **Region (ref: Western)** | Reference |  |
| Central | 0.96 | [0.81,1.14] |
| Eastern | 0.97 | [0.79,1.17] |
| *Peer influence* |  |  |
| **Connectedness with mother (ref: Can never/sometimes discuss personal matters with mother)** | Reference |  |
| Can always/most times discuss personal matters with mother | 0.93 | [0.74,1.17] |
| **Connected with father (ref: Can never/sometimes discuss personal matters with father)** | Reference |  |
| Can always/most times discuss personal matters with father | 0.96 | [0.78,1.18] |
| **Community peers’ attitude on gender roles (ref: Non-egalitarian)** | Reference |  |
| Somewhat egalitarian | 2.37^**^ | [1.71,3.28] |
| Egalitarian | 5.27^**^ | [3.35,8.28] |
| ***Social connectivity*** |  |  |
| **Watches TV at least once a week (ref: No)** | Reference |  |
| Yes | 1.21 | [0.96,1.52] |
| **Accesses internet at least once a week (ref: No)** | Reference |  |
| Yes | 0.94 | [0.79,1.11] |
| **Member of any youth club/social organization (ref: No)** | Reference |  |
| Yes | 1.51^**^ | [1.26,1.82] |
| **Participation in any program focused on adolescents (anytime in the last 3 years) (ref: No)** | Reference |  |
| Yes | 1.42^*^ | [1.05,1.90] |
| Observations | 5399 |  |

^*^ *p* < 0.05, ^**^ *p* < 0.01
